# Timing of oomycete-specific fungicide application impacts the efficacy against fruit rot disease in arecanut

**DOI:** 10.3389/fpls.2023.1237795

**Published:** 2023-09-14

**Authors:** Patil Balanagouda, Sandip Shil, Shankarappa Sridhara, R. Thava Prakasa Pandian, Shivaji Hausrao Thube, Vinayaka Hegde, Shaban R. M. Sayed, Rayan Casini, Hanumappa Narayanaswamy

**Affiliations:** ^1^ Department of Plant Pathology, Keladi Shivappa Nayaka University of Agricultural and Horticultural Sciences, Shivamogga, Karnataka, India; ^2^ Division of Social Sciences, ICAR-Central Plantation Crops Research Institute, Research Centre, Jalpaiguri, West Bengal, India; ^3^ Center for Climate Resilient Agriculture, Keladi Shivappa Nayaka University of Agricultural and Horticultural Sciences, Shivamogga, Karnataka, India; ^4^ ICAR-Central Plantation Crops Research Institute, Regional Station, Vittal, Karnataka, India; ^5^ ICAR-Central Institute for Cotton Research, Nagpur, Maharashtra, India; ^6^ Division of Crop Protection, ICAR-Central Plantation Crops Research Institute, Kasaragod, Kerala, India; ^7^ Department of Botany and Microbiology, College of Science, King Saud University, Riyadh, Saudi Arabia; ^8^ School of Public Health, University of California, Berkeley, Berkeley, CA, United States

**Keywords:** fruit rot disease, fungicide efficacy, application timing, GLMM, oomycete-specific fungicides

## Abstract

Fungicidal application has been the common and prime option to combat fruit rot disease (FRD) of arecanut (*Areca catechu* L.) under field conditions. However, the existence of virulent pathotypes, rapid spreading ability, and improper time of fungicide application has become a serious challenge. In the present investigation, we assessed the efficacy of oomycete-specific fungicides under two approaches: (i) three fixed timings of fungicidal applications, i.e., pre-, mid-, and post-monsoon periods (EXPT1), and (ii) predefined different fruit stages, i.e., button, marble, and premature stages (EXPT2). Fungicidal efficacy in managing FRD was determined from evaluations of FRD severity, FRD incidence, and cumulative fallen nut rate (CFNR) by employing generalized linear mixed models (GLMMs). In EXPT1, all the tested fungicides reduced FRD disease levels by >65% when applied at pre- or mid-monsoon compared with untreated control, with statistical differences among fungicides and timings of application relative to infection. In EXPT2, the efficacy of fungicides was comparatively reduced when applied at predefined fruit/nut stages, with statistically non-significant differences among tested fungicides and fruit stages. A comprehensive analysis of both experiments recommends that the fungicidal application can be performed before the onset of monsoon for effective management of arecanut FRD. In conclusion, the timing of fungicidal application based on the monsoon period provides better control of FRD of arecanut than an application based on the developmental stages of fruit under field conditions.

## Introduction

Fruit rot disease (FRD) is a highly destructive disease of arecanut (*Areca catechu* L.) and other palms such as the coconut and oil palm and has become a critical concern to arecanut production in the last two decades in India (Jose et al., 2008; Chowdappa et al., 2014; Balanagouda et al., 2021). FRD was first documented in the former Mysore state (Butler, 1906), and subsequent occurrences were recorded in the coastal areas of Karnataka and the Malabar region of Kerala in India (Coleman, 1910). Presently, this disease seems to occur in all the arecanut-growing regions that receive considerable monsoon rains during the *Kharif* season (Sarma et al., 2002). FRD is caused by the *Phytophthora* species complex of which *P. meadii* (Sastry and Hedge, 1985; Balanagouda et al., 2022a) predominantly occur on arecanut plantations in India. *P. palmivora* (Das and Cheeran, 1986), *P. heveae* (Chowdappa et al., 2002), and *P. arecae* (Pethybridge, 1913) are also frequently isolated from FRD-infected samples in India.

Arecanut, a hardy crop, is highly vulnerable to FRD which causes considerable economic losses to the growers and results in substantial qualitative and quantitative losses (Jose et al., 2008). FRD infection has been reported to cause 10%–90% of yield losses, leading to the death of 10%–15% of palms due to bud/crown rot in arecanut plantations (Koti Reddy and Anandaraj, 1980). The presence of highly virulent strain/s and the quick spreading ability of pathogens coupled with improper application timing of chemical measures under field conditions have become a major challenge for effective management of FRD of arecanut.

FRD remains a major challenge and threat to arecanut growers due to its persistent and fast-spreading nature, perpetuation, and rapid development under favorable weather conditions (Balanagouda et al., 2022b). The occurrence of FRD has increased over the years due to the accumulation of inoculum in endemic regions during the onset of monsoon rains, combined with lower temperatures and higher relative humidity (Coleman, 1910; Balanagouda et al., 2022b). FRD typically occurs 15–20 days following the first showers of the southwest monsoon (May–June) and persists until the end of the rainy season (August–September). In seasons where rainfall is sporadic and irregular, there have been instances where a gap of 40–50 days has been observed between the first monsoon rain and the onset of fruit rot (Marudarajan, 1950; Anandaraj and Saraswathy, 1986; Saraswathy, 1994).

In the last two decades, considerable attempts have been made to manage FRD through the integration of multiple strategies (Hegde, 2015; Narayanaswamy et al., 2017; Gangadhara Naik et al., 2019; Balanagouda et al., 2023), including preventive fungicide spraying, the use of fertilizer-amended fungicidal briquettes, the exploitation of bio-agents, and phytosanitation (removal of infected fallen nuts). Despite this, the use of fungicides remains a key option for managing FRD under field conditions (Chowdappa et al., 2002; Ravikumar et al., 2019), but its efficacy remains highly inconsistent due to various limitations. This variability may be attributed to mode of action, formulation type, and timing of application of fungicides.

Since the first report of FRD in 1906, copper-based and phenylalanine fungicidal molecules have been widely used to manage the disease in field conditions (Coleman, 1910; Sastry and Hedge, 1985; Saraswathy, 2004; Prathibha et al., 2016), and their efficacy was not consistent between the various experiments. To date, no studies have evaluated the combined impact of application timing and fruit developmental stage on the incidence of arecanut FRD. Although few studies stated about the prophylactic application of fungicides before onset and the mid-monsoon period (twice or thrice in a season) provide better management of FRD (Lokesh et al., 2014; Narayanaswamy et al., 2017), no systematic investigations have demonstrated the effect of application timing on fungicidal efficacy in the management of FRD.

Therefore, the present study was conducted to determine the effect of the timing of fungicide application and the developmental stages of the arecanut fruit on the efficacy of fungicidal active ingredients in reducing the incidence and severity of FRD in arecanut. To the best of our knowledge, this is the first study focusing on the fixed timing of fungicide application and fruit stage to combat FRD under field conditions, to improve arecanut yield and productivity.

## Materials and methods

### Field experiments

Two experiments were established in this study. Experiment 1 (EXPT1) was conducted at the Agricultural and Horticultural Research Station (AHRS) in Thirthahalli, which is part of the University of Agricultural and Horticultural Sciences in Shivamogga, India. The location of AHRS is at 13.41600° N latitude and 75.13480° E longitude. A second experiment, EXPT2, was conducted on a farmer’s plantation at Bhandigadi Village of Koppa Taluk which belongs to Chikmagalur district, Karnataka State, India, located between 13.5575°N latitude and 75.2673°E longitude. The selected fungicidal products for the study are commercially available and specifically labeled for controlling FRD and other *Phytophthora* diseases in India. They are commonly used in arecanut plantations to prevent fruit and foliar diseases caused by fungal-like organisms.

EXPT1 and EXPT2 were conducted in 2018 and 2019 on the FRD highly susceptible areca cv. Mangala variety. During 2018 and 2019, a total of 12 fungicides specific to oomycete as individual active ingredients or premixed formulations (**Table 1**) were evaluated and compared with an untreated control in their efficacy to control FRD. Each of 12 different fungicides commercially labeled for oomycetes management was applied at three fixed timings during the southwest monsoon period (Narayanaswamy et al., 2017): (i) *pre-monsoon*—application of fungicides before the start of the monsoon season; (ii) *mid-monsoon*—fungicides were sprayed in the second half of July (45 days after pre-monsoon application), and (iii) *fag-end or late monsoon*—fungicides were applied in the last week of August. The experiment was laid out in a randomized complete block design (RCBD) with three replications. The experimental plantation was situated on a slope of 5%–6% with a distance of 2.7 m between rows. There were 50 palms evaluated in each treatment per replication (in total 1,800 palms), and the untreated palms were replaced regularly to avoid the spread of the disease.

**Table 1:** Details of the commercial fungicides evaluated under the experiments in 2018 and 2019 in India.

Common name of active ingredient fungicide active ingredient/s	% Active ingredient	Formulation	Commercial name	Producer	FRAC code ^a,b^
**Famoxadone + Cymoxanil**	38.7 (2.5 mL)	SC	Equation Pro	DuPont	CAO (27)
**Kresoxymethyl**	44.3 (1 mL)	SC	Ergon	Rallis India Ltd	QoI (11)
**Mandipropamid**	23.4 (5 mL)	SC	Revus	Syngenta	CAA (40)
**Dimethomorph + Mancozeb**	80 (2 mL)	WP	Acrobat	Syngenta	CAA (40) DCR (M03)
**Ametoctradin + Dimethomorph**	55.25 (2 mL)	SC	Zampro	BASF	QOSI (45) CAA (40)
**Cymoxanil + Mancozeb**	72 (2 g)	WP	Curzate	DuPont	CAO (27) DCR (M03)
**Iprovalicarb +Propineb**	66.75 (2.5 g)	WP	Melody Duo	Bayer	CAA (40) DCR (M03)
**Metiram + Pyraclostrobin**	55 (2.5 g)	WG	Clutch	PI Industries	DCR (M03) QoI (11)
**Copper hydroxide**	77 (3 g)	WP	Kocide	DuPont	Inorganic (M01)
**Metalaxyl + Mancozeb**	72 (2.5 g)	WP	Ridomil Gold	Syngenta	PA (04) DCR (M03)
**Bordeaux mixture**	24.3 (1%)	SL	Manual	–	Inorganic (M01)
**Fosetyl-Al**	80 (3 g)	WP	Aliette	Bayer	Phosphonate (P07 (33))

a FRAC Code List (2021), Fungicide Resistance Action Committee.

b CAO, Cyanoacetamide-oxime; CAA, carboxylic acid amides; QOI, quinone outside inhibitors; DCR, dithiocarbamates and relatives; QOSI, quinone outside inhibitors—stigmatellin binding type; PA, phenyl amides.

c R, respiration; CWB, cell wall biosynthesis; MSA, multisite action; NAM, nucleic acid metabolism; HPDI, host plant defense induction; UKN, unknown; MET, mitochondrial electron transport.

In EXPT2, the fungicides were applied at three developmental stages of arecanut fruit: (i) *button stage*—fungicides were applied to tender, immature green arecanut during June–July months; (ii) *marble stage*—fungicides were applied to slightly mature, hard-surfaced arecanut during August–September, and (iii) *premature stage*—fungicides were applied to rough, premature, larger-sized arecanut in October. The experimental setup was similar to those in EXPT1 for both years.

### Application of the fungicides

A portable rocket sprayer^®^ (model RHSD-0131 with heavy duty, Maico Pipe Industries, Gujarat, India) fitted with an adjacent cone jet tip and one flexible nozzle was used to apply the fungicides in both experiments. The sprayer had an ABS plastic nozzle (Maico Pipe Industries, Gujarat, India) and the potential to dispense the spray to heights of 25–30 ft as well as 75–80 gallons on a single battery charge. The nozzle had an operational pressure of approximately 10–15 bars and a heavy delivery set (5 m long) with a nut nipple connection. The pump barrel and pressure chamber were attached for ease of use by hand, and the spray lance was equipped with a triple-action brass nozzle that could spray fungicides up to a height of 20–30 ft. Each fungicidal treatment was applied using 600 L/ha of water along with premixed wetting/adhesive agent Indetran to ensure proper adhesion of applied fungicides on the fruit surfaces.

### Assessment of disease variables

In both years of experimentation, variables related to the disease such as the incidence of FRD, the severity of FRD, and cumulative fallen nut rate (CFNR) were recorded and calculated. FRD incidence was computed as the proportion of infected palms to healthy palms. The severity of FRD was determined as the percentage of the arecanut bunch surface showing FRD symptoms and was estimated using a standard 0–6 scale (Sastry and Hedge, 1987). CFNR was calculated as the average number of fallen nuts due to FRD infection per palm. The FRD incidence, FRD severity, and CFNR were evaluated by examining dropped nuts on the ground in each treatment (total of 1,800 palms were evaluated) including untreated control and infected nuts present on the bunch, which has not been considered for rating.


$$FRD\;incidence=\;\frac{\text{Number of infected palms}}{\text{Total number of observed palms}}\;\times 100$$

$$FRD\;severity=\;\frac{\text{Sum of numerical ratings}}{\text{Total number of plants observed }\times \text{Maximum grade}}\;\times 100$$

CFNR = Average number of fallen nuts due to FRD infection/palm

**Figure 1:**
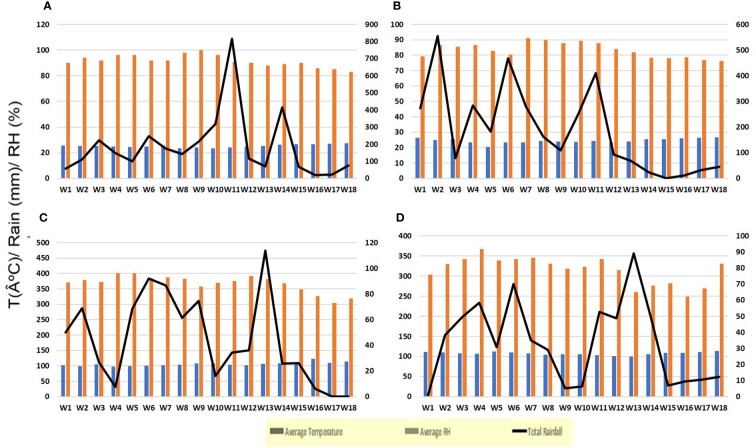
Weather variables recorded from June to September in the experimental plantations at AHRS, Thirthahalli (EXPT-1), and Bhandigadi village (EXPT-2); **(A)** year 2018 (EXPT-1), **(B)** year 2019 (EXPT-1), **(C)** year 2018 (EXPT-2), **(D)** year 2019 (EXPT-2). The figure parenthesis represents the standard weekly mean temperature (blue bards; in degrees Celsius); average relative humidity (RH; in light orange bars; in percentage); and total rainfall (in black lines; in millimeters).

During the June to September period, the average temperature (°C), relative humidity (%), and total rainfall (mm) were recorded every week for both experiments (**Figure 1**). The data were recorded at the Thirthahalli and Sringeri Agricultural and Horticultural Research Station (AHRS) meteorological station, which are maintained by the State Agricultural Departmental Service (SADS) in the Indian provinces of Thirthahalli and Sringeri and affiliated with the Indian Meteorological Department (IMD) in Pune, India.

### Confirmation of fruit rot pathogen in an experimental plot

To confirm the identity of the FRD pathogen that exists in the experimental field, symptomatic nuts were subjected to cultural and molecular characterization of the associated pathogen. A total of 20 symptomatic nuts from 10 different palms were collected, washed under running tap water, cut into small pieces, surface sterilized in 2% NaOCl for 60 s, rinsed in distilled water four times, and air dried. A small piece of infected tissue was placed on carrot agar (CA) plates and incubated at 24 ± 2° C for 6–8 days (Ribeiro, 1978; Pandian et al., 2021). Cultural and morphological observations of the isolated pathogen were carried out as per the description given by Balanagouda et al. (2022a). Total genomic DNA was isolated from the pure culture of the pathogen following the CTAB method with minor modifications (Pandian et al., 2018). Molecular amplification of the internal transcribed spacer region of ribosomal DNA using ITS1 (5′-TCC GTA GGT GAA CCT GCG G-3′) and ITS4 (5′-TCC TCC GCT TAT TGA TAT GC-3′) primers was carried out to confirm the identity of the pathogen (White et al., 1990). The amplified product was evaluated with electrophoresis (Major Science, USA) using 1.2% agarose gel (Sambrook and Russell, 2001). The PCR- amplified products were purified using a PCR purification kit (Geneaid, Taiwan), and purified products were sent for Sanger sequencing (AgriGenome Labs Pvt. Ltd., Cochin, India). Obtained sequences were aligned using BioEdit (biological sequence alignment editor —Tom Hall, http://www.mbio.ncsu.edu/BioEdit/bioedit.html) and compared with the available sequences in the NCBI (http://www.ncbi.nlm.nih.gov/BLAST) and BOLD databases (http://www.boldsystems.org/). The multiple-sequence alignment was performed by using the Clustal W program with the pathogen sequence along with available sequences (Thompson et al., 1994). The end-trimmed pathogen sequence was deposited in the NCBI database.

### Data analysis

A mixed effect model was applied to the generated data and was analyzed by using R software 3.6.0 (R Core Team, 2020). Data on disease variables, *viz*., FRD incidence, FRD severity, and CFNR, from both experiments were included as explanatory variables in generalized linear mixed model (GLMM) analyses. Using the “lme4” package’s function glmer, the GLMM was run for all the variables with a Gaussian distribution and logit link function (Madden et al., 2002; Bates et al., 2015). Fungicides (FUNG) and application timing (TIME) were regarded as fixed effects in EXPT1, whereas fruit/nut spraying stage (STAGE) and fungicides (FUNG) were considered as fixed effects in EXPT2. While disease pressure in the experimental years (YEAR) was seen as a subset of the whole population and disease varied with environmental variables between the years, the experimental years (YEAR) were treated as random effects.

In EXPT1, four models were used to analyze each dataset: one with only YEAR as a random factor, another with FUNG as a fixed factor and YEAR as a random effect, a third with FUNG + TIME as a fixed factor and YEAR as a random factor, and a fourth with the interaction FUNG×TIME as a fixed effect and YEAR as a random factor. Similar models were also applied to the EXPT2 dataset, with the exception of the fruit/nut stage (STAGE) variable. The four models are (i) only YEAR as a random factor, (ii) FUNG as a fixed factor and YEAR as a random effect, (iii) FUNG + STAGE as a fixed factor and YEAR as a random factor, and (iv) the interaction between FUNG and STAGE as a fixed effect and YEAR as a random factor. Based on the lowest Akaike’s information criterion (AIC) value and the probability level of the *chi-square test* performed using the function ANOVA, the best-fit model was selected (Quinn and Keough, 2011; Crawley, 2013). The standardized Pearson residuals for the various levels of each factor were visually examined in order to assess the model’s goodness of fit. The “DHARMa” package tests *dispersion* and simulates the residual’s function to evaluate the value of the dispersion and residuals (Hartig, 2021).

Three-factor ANOVA was conducted for EXPT1 and EXPT2, and using the *F*-statistic, we calculated the amount of variation accounted by each predictor relative to the left-over error variance. Furthermore, the influence of the tested fungicides on FRD control over the years was computed and compared with model parameters such as *F*-value, *P*-value, and standard error by using R software 3.6.0 with the AGRICOLAE package.

## Results

### Symptoms of fruit rot

Fruit rot disease (FRD) of arecanut is characterized by rotting and extensive shedding of the immature nuts which lie scattered near the base of the palm. Initial symptoms appear as dark green/yellowish water-soaked lesions on the nut surface near the perianth (calyx). Later, the lesions on the fruits gradually spread covering the whole surface before or after shedding which consequently rot. White mycelial mass enveloped on entire surface of the fallen nuts, and as the disease advances, the fruit stalks and the axis of the inflorescence were rotten and dried.

### Morphological and molecular characterization of pathogen

The pathogen associated with FRD in the experimental field was identified as Phytophthora meadii. The PCR amplification of the ITS gene sequence resulted in amplification of a 641-bp nucleotide, which was 100% nucleotide similarity with P. meadii with GenBank accession No. LC076469, and the amplified gene sequences were submitted to GenBank (ON999172).

### Fixed timing of fungicide application against FRD (EXPT1)

During the southwest monsoon period of 2018 and 2019, the weather conditions were mostly rainy with intermittent sunny spells (as shown in **Figures 1A, B**). The average FRD severity in the untreated control for the years 2018 and 2019 was 30.33% and 40.93%, respectively. The FRD incidence in the untreated control for the same years was 14.5% and 21.6%, respectively. The cumulative fallen nut rate (CFNR) in the untreated check ranged from 25 to 115 infected fallen nuts per palm (data not shown).

According to the GLMM, the interaction between fungicides and application timings considerably impacted the severity, incidence, and CFNR of FRD. The GLMM’s 1.4, 1.8, and 1.12, which represented the interaction impact of FUNG × TIME, had the lowest AIC and deviance values at significance, showing that the interaction effect of two factors determined the model’s explanatory variability (**Table 2**). These models’ dispersion and residuals showed that the expected and actual values generally agreed.

**Table 2:** Generalized linear mixed models (GLMMs) fit the data gathered from the experiment conducted at AHRS, Thirthahalli, Karnataka, India, for the control of FRD at fixed timing of fungicidal application (EXPT1).

Experiment^1^	Variable	Model^2^	Factors^3^	AIC^4^	Deviance	Chisq	*P(>Chisq)*
**EXPT1**	FRD severity	1.1	(1|YEAR)	1,740	1,734	–	–
	FRD severity	1.2	FUNG + (1|YEAR)	1,670	1,640	193.3	<0.001
	FRD severity	1.3	FUNG + TIME + (1|YEAR)	1,249	1,215	425.3	<0.001
	FRD severity	1.4	FUNG_TIME + (1|YEAR)	1,111	1,029	186.0	<0.001
**EXPT1**	FRD incidence	1.5	(1|YEAR)	1,667	1,661	–	–
	FRD incidence	1.6	FUNG + (1|YEAR)	1,598	1,568	192.6	<0.001
	FRD incidence	1.7	FUNG + TIME + (1|YEAR)	1,154	1,120	448.1	<0.001
	FRD incidence	1.8	FUNG_TIME + (1|YEAR)	1,141	1,059	60.8	<0.001
**EXPT1**	CFNR	1.9	(1|YEAR)	2,444	2,438	–	–
	CFNR	1.10	FUNG + (1|YEAR)	2,374	2,344	193.4	<0.001
	CFNR	1.11	FUNG + TIME + (1|YEAR)	1,953	1,919	425.4	<0.001
	CFNR	1.12	FUNG_TIME + (1|YEAR)	1,815	1,733	185.9	<0.001

^1^ EXPT1, fungicides were applied at three fixed timings of the southwest monsoon period; pre-monsoon (before the onset of rain), mid-monsoon (second fortnight of July), and fag-end of monsoon (last week of August) as per the earlier reports (Narayanaswamy et al., 2017). ^2^Models for all the variables were run with a Gaussian distribution and a logit-link function. ^3^ Fungicides (FUNG) and timing of application (TIME) were considered as fixed factors, whereas years (YEAR) were considered as random effects. ^4^ AIC, Akaike’s information criterion; Deviance, minus the maximized log-likelihood; Chisq, Chi test and the associated probabilities (P value) when comparing the models with the same dataset.

**Figure 2:**
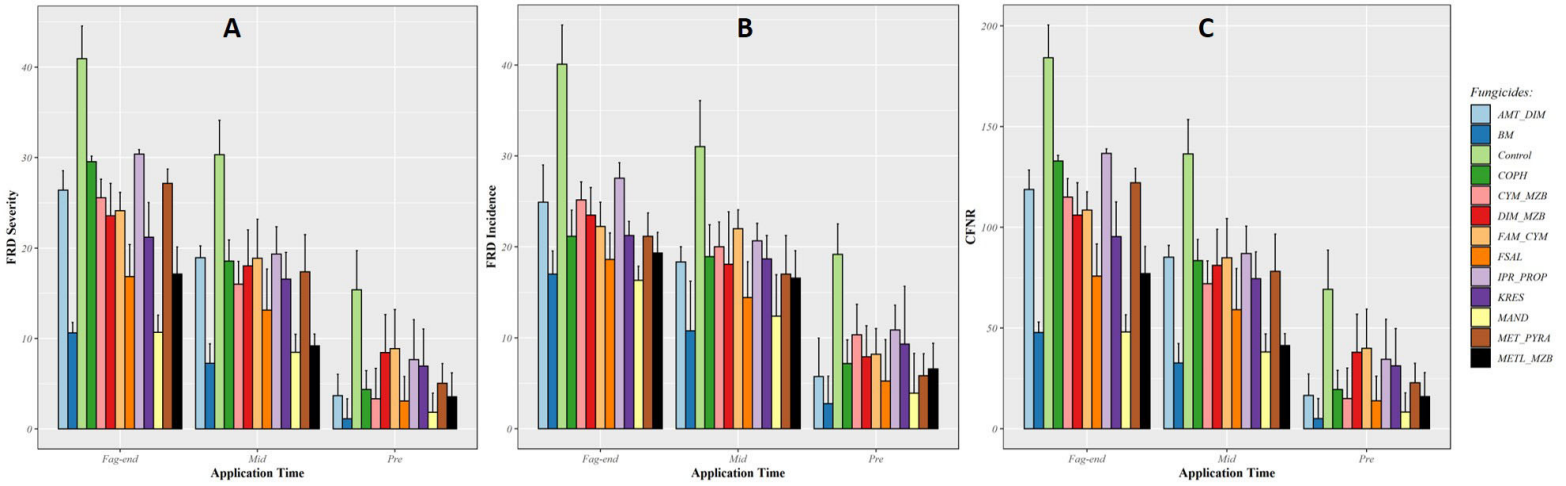
Efficacy of fungicide treatments applied at three fixed timings in reducing fruit rot disease (FRD) severity **(A)**, incidence **(B)**, and CFNR **(C)** in EXPT1. Fungicides were applied at three fixed timings of the southwest monsoon period; pre-monsoon (before the onset of rain), mid-monsoon (second fortnight of July), and fag-end of monsoon (last week of August).

Based on the findings from these models, the use of fungicides such as Mandipropamid, Bordeaux mixture, Metalaxyl + Mancozeb, Fosetyl-Al, and other copper-based products led to a significant decrease in FRD severity, FRD incidence, and CFNR when applied prior to the start of monsoon (**Figure 2**). The effectiveness of these fungicides was diminished when applied during mid-monsoon and at the end of the rainy season due to persistent rainfall. On the other hand, the average FRD control efficiency of Mandipropamid (87%), Bordeaux mixture (76.34%), Metalaxyl + Mancozeb (69.95%), and Fosetyl-Al (61.02%) was higher when applied before the beginning of the monsoon. The use of these fungicidal molecules resulted in significant reductions in the incidence, severity, and CFNR of FRD, even when applied during mid-monsoon and at the end of the rainy season when compared with untreated control (as shown in **Figures 2A-C**).

Under field conditions, all the fungicides showed a significant reduction in FRD (*P* < 0.05) compared with untreated control, when applied at fixed timings. The impact of the fungicides on FRD severity, incidence, and CFNR was found to be not significant when applied at the end of the monsoon season, due to varying degrees of efficacy among the fungicides and lack of statistical significance in the timing of application. Experiment 1 demonstrated that applying the fungicides before the start and during the middle of the monsoon season led to better control of FRD with higher efficacy and reduced disease occurrence compared with application at fag-end of the monsoon.

The results from the ANOVA (**Table 3**) suggested that the efficacy of fungicides was found to be significant (*P* < 0.01) while controlling for FRD severity, incidence, and CFNAR under field conditions. Similarly, other variables such as time of fungicidal application and their interaction with fungicidal potential had significant differences (*P* < 0.01) on dependent variables FRD severity, FRD incidence, and CFNAR. Hence, the timing of fungicidal application has a crucial role in deciding the efficiency of evaluated fungicides in controlling FRD under field conditions.

**Table 3:** Analysis of variance (ANOVA) for the effect of different fungicides, time of application, and their interaction on FRD severity, incidence, and CFNR in arecanut.

Sources of variation	df	Sum of squares	Mean sum of squares	F value	P value
FRD severity					
Fungicides	12	7,345.9	612.2	111.6	0.001
Time of application	2	12,462.7	6,231.3	1,136.1	0.008
Fungicides × time of application	24	1,307.7	54.5	9.93	0.002
FRD incidence					
Fungicides	12	5,307.5	442.3	71.1	0.001
Time of application	2	9,250.6	4,625.3	743.8	0.006
Fungicides × time of application	24	361.6	15.1	2.42	0.001
CFNR					
Fungicides	12	148,800	12,400	111.6	0.008
Time of application	2	252,291	126,146	1,135.7	0.002
Fungicides × time of application	24	26,458	1,102	9.92	0.005

### Application of fungicides at different fruit/nut stages (EXPT2)

EXPT2 revealed that the weather conditions from June to July 2018 were slightly drier than in 2019, with a rainfall of 650 mm in 2018 and 840 mm in 2019 (as seen in **Figures 1C, D**). The severity, incidence, and CFNR of FRD in the control plots were higher in 2019 compared with 2018. The average severity of FRD was 20.2% in 2018 and 24.5% in 2019, whereas the incidence was 15.8% in 2018 and 19.7% in 2019. The CFNR was 56 infected nuts per palm in 2018 and 65 infected nuts per palm in 2019 (the data are not shown).

Similar to what was observed in EXPT1, experiment 2 (EXPT2) also showed that the severity, incidence, and CFNR of FRD were significantly influenced by the interaction between the fungicides and the different stages of their application. Models 2.4, 2.8, and 2.12, which accounted for the interaction impact of FUNG × STAGE, showed the lowest AIC and deviation values with a significance of *P <* 0.001 (as seen in **Table 4**) . These models also had a good agreement between the expected and observed data in terms of dispersion and residual values.

**Table 4:** Generalized linear mixed models fit the data collected from an experiment carried out at Bhandigadi village, Koppa, Karnataka, India, for the control of FRD using fungicidal spraying at different fruit/nut stages (EXPT2).

Experiment^1^	Variable	Model^2^	Factors^3^	AIC^4^	Deviance	Chisq	*P(>Chisq)*
**EXPT2**	FRD severity	2.1	(1|YEAR)	1,463	1,457	–	–
	FRD severity	2.2	FUNG + (1|YEAR)	1,358	1,328	129.4	<0.001
	FRD severity	2.3	FUNG + STAGE + (1|YEAR)	1,087	1,053	274.4	<0.001
	FRD severity	2.4	FUNG_ STAGE + (1|YEAR)	1,090	1,008	45.5	<0.001
**EXPT2**	FRD incidence	2.5	(1|YEAR)	1,451	1,445	–	–
	FRD incidence	2.6	FUNG + (1|YEAR)	1,341	1,311	133.3	<0.001
	FRD incidence	2.7	FUNG + STAGE + (1|YEAR)	1,082	1,048	263.3	<0.001
	FRD incidence	2.8	FUNG_ STAGE + (1|YEAR)	1,088	1,006	42.4	<0.001
**EXPT2**	CFNR	2.9	(1|YEAR)	2,112	2,106	–	–
	CFNR	2.10	FUNG + (1|YEAR)	2,007	1,977	129.4	<0.001
	CFNR	2.11	FUNG + STAGE + (1|YEAR)	1,736	1,702	274.3	<0.001
	CFNR	2.12	FUNG_ STAGE + (1|YEAR)	1,739	1,657	45.5	<0.001

^1^ EXPT2, fungicide products were applied at different fruit growth stages, i.e., button, marble, and premature nut stages. ^2^ Models for all the variables were run with a Gaussian distribution and a logit-link function. ^3^ Fungicides (FUNG) and timing of application (TIME) were considered as fixed factors, whereas years (YEAR) were considered as random effects. ^4^ AIC, Akaike’s information criterion; Deviance, minus the maximized log-likelihood; Chisq, Chi test and the associated probabilities (P value) when comparing the models with the same dataset.

**Figure 3:**
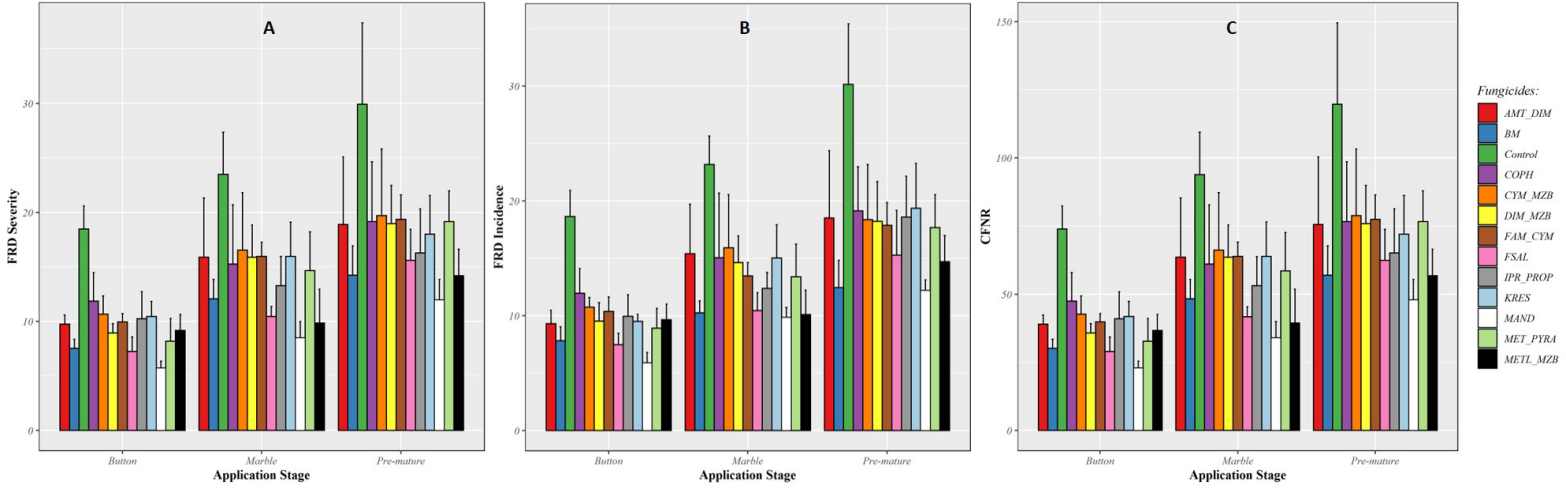
Efficiency of fungicidal products applied at three fixed fruit/nut stages in controlling fruit rot disease (FRD) severity **(A)**, incidence **(B)**, and CFNR **(C)** in EXPT2. Fungicide products were applied at different fruit growth stages, *i.e*., button, marble, and premature nut stages.

In EXPT2, the results of the models showed that the fungicides with multiple actions reduced the severity, incidence, and CFNR of FRD but were not statistically significant compared with the untreated control (*P* > 0.001). However, the Bordeaux mixture, Mandipropamid, copper oxychloride, Metalaxyl + Mancozeb, and Fosetyl-Al fungicides showed higher efficacy against FRD when applied under field conditions at different fixed fruit stages (as seen in **Figure 3**). There were no significant (*P* < 0.05) differences among the fungicides tested when applied at fixed fruit/nut stages, such as button, marble, and premature stages (EXPT2).

The severity of FRD was reduced by more than 60% compared with the untreated control when the fungicides were applied at the button stage. Although the efficacy of the fungicides was reduced when applied at the marble and premature stages, the difference in FRD reduction was not statistically significant (as shown in **Figure 3A**). The average efficacy of fungicides in reducing FRD incidence ranged from 35% to 50% when applied at the button stage. The efficacy was lower when the fungicides were applied at the marble and premature stages compared with when they were applied at the button stage (as shown in **Figure 3B**). A similar trend was also observed for CFNR. Fungicides applied at the button stage significantly reduced CFNR compared with when they were applied at the marble and premature stages (as shown in **Figure 3C**), and there were no statistically significant differences in CFNR values among the tested fungicides.

Analysis of variance clearly indicated that the stage of fungicidal application does not significantly predict their interaction with fungicide efficacy (*P* > 0.05), although there was a significant difference observed on efficacy of fungicides in controlling FRD (*P* < 0.05) and stage of fungicidal application (*P* > 0.05). However, stage of fungicidal application did not significantly contribute in controlling for FRD compared with the timing of application (**Table 5**).

**Table 5:** Analysis of variance (ANOVA) for the effect of different fungicides, stage of application, and their interaction on FRD severity, incidence, and CFNR in arecanut.

Sources of variation	df	Sum of squares	Mean sum of squares	F value	P value
FRD severity					
Fungicides	12	2,867.2	238.9	47.9	0.01
Stage of application	2	2,662.0	1,330.9	267.1	0.04
Fungicides × stage of application	24	209.6	8.73	1.75	0.52
FRD incidence					
Fungicides	12	2,792.7	232.7	46.8	0.01
Stage of application	2	2,439.6	1,219.8	245.7	0.45
Fungicides × stage of application	24	193.05	8.04	1.62	0.55
CFNR					
Fungicides	12	45,868	3,822.3	47.9	0.01
Stage of application	2	42,589	21,294.3	266.9	0.04
Fungicides × stage of application	24	3,355	139.8	1.75	0.62

### Influence of years and moment of fungicidal application on FRD control

The analysis of variance (ANOVA) indicated the influence of the fungicides, timing of the spray over the years on FRD severity, incidence, and CFNR. Significant differences were observed with respect to the timing of the fungicide spray as well as different fungicides and their interactions (**Table 6**). Even though there exists a differential response for the application timings and fungicides over the years, the interaction effect of Y **×** T **×** F was found to be non-significant. Hence, the timing of the fungicidal spray plays an important role in reducing the FRD severity and incidence with decreased CFNR.

**Table 6:** Influence of fungicides, years, and moment of fungicidal application on FRD controlling ability of fungicides under field conditions (EXPT1).

Source of variation	df	FRD severity		FRD incidence		CFNR	
		F value	Significance	F value	Significance	F value	Significance
Replications	2	1.2996	ns	50.4992	***	1.2996	ns
Year (Y)	1	133.4771	***	321.9142	***	133.4771	***
Timing (T)	2	1,183.7051	***	1,328.7297	***	1,183.7051	***
Fungicides (F)	12	116.2855	***	127.0580	***	116.2855	***
Y × T	2	4.8997	**	11.1725	***	4.8997	**
Y × F	12	1.4313	ns	1.8898	*	1.4313	ns
T × F	24	10.3504	***	4.3283	***	10.3504	***
Y × T × F	24	0.7728	ns	1.9379	**	0.7728	ns

ns, non-significant.

Significant codes: *** 0.001. ** 0.01, * 0.05.

ANOVA suggested the lesser influence of the fungicides, and stage of the fungicidal application over the years on FRD severity, incidence, and CFNR (**Table 7**). Although fungicidal efficacy was found to be significant, the stage of fungicide spray, years of evaluation, and their interactions were non-significant. The differential response of fungicides over the years was observed, and the interaction effect of Y **×** S **×** F was found to be non-significant. However, the stage of fungicidal application did not considerably influence on FRD severity, incidence, and CFNR under field conditions.

**Table 7:** Influence of fungicides, years, and stage of fungicidal application on FRD controlling ability of fungicides under field conditions (EXPT2).

Source of variation	df	FRD severity		FRD incidence		CFNR	
		F value	Significance	F value	Significance	F value	Significance
Replications	2	2.5798	ns	4.3938	*	2.5798	ns
Year (Y)	1	2.4212	ns	1.4537	ns	2.4212	ns
Stage (S)	2	0.5060	ns	0.1004	ns	0.5060	ns
Fungicides (F)	12	7.9768	***	9.3543	***	7.9768	***
Y × S	2	0.0467	ns	0.0063	ns	0.0467	ns
Y × F	12	0.1265	ns	0.1272	ns	0.1265	ns
S × F	24	0.1007	ns	0.1120	ns	0.1007	ns
Y × S × F	24	0.0540	ns	0.0432	ns	0.0540	ns

ns, non-significant.

Significant codes: *** 0.001. ** 0.01, * 0.05.

## Discussion

In this study, we evaluated the effectiveness of two overall fungicide application methods for FRD management in arecanut under field conditions. One approach was a calendar-based strategy, commonly followed by arecanut growers in India, where fungicides are applied once before the onset of monsoon (May last week) to prevent FRD infection (Narayanaswamy et al., 2017; Gangadhara Naik et al., 2019; Ravikumar et al., 2019; Balanagouda et al., 2023). This approach involves assessing the efficacy of fungicides as preventative (preinfection) and curative (postinfection) measures and spraying fungicides based on the risk of infection and their pre- and postinfection properties, making it a novel technique. This method follows the principles of integrated disease management (IDM), as it is based on knowledge of the pathogen’s biology, environmental factors, and fungicidal action mechanisms. The second strategy, which is not commonly used for arecanut FRD management, was based on administering fungicides at three separate fruit/nut stages and is widely used in other crops (González-Domínguez et al., 2021). This approach optimizes the fungicide usage, reduces the number of applications, and helps to prevent fungicide resistance if used correctly (Rossi et al., 2012; Rossi et al., 2019).

The results of the EXPT1 showed that the timing of fungicide application had a significant impact on its efficacy in reducing FRD levels and CFNR. A reduction of more than 65% was observed with significant differences among the evaluated timing of application. In agreement with the previous studies, we found that applying fungicides prior to the onset of monsoon is an effective measure in reducing FRD (Hegde, 2015; Pande et al., 2016), and a meta-analysis of 22 studies concluded that preventive application of fungicides made a significant difference in their efficacy (Balanagouda et al., 2022c). Applying fungicides as a preventive measure can significantly reduce FRD infection, and its application as a curative measure can also help to stop the spread of FRD during the mid-monsoon period. However, a significant reduction in the efficacy of fungicide molecules when applied at the end of the monsoon is attributed to improper application, and dispersal of fungicidal solution due to heavy rainfall coupled with congenial abiotic factors and a heavy load of inoculum.

The results of EXPT2 showed that applying fungicides at different fruit/nut stages had little impact on their efficacy, and this was statistically insignificant. When fungicides were applied at different nut stages, their effectiveness in managing the FRD was lower. These results support that the time of fungicide application, rather than the stage of the arecanut, determines its effectiveness as a preventive or curative measure against FRD infection. Therefore, the experiment results suggested that using a timing-based strategy, rather than a phenology-based strategy, is a more effective way to combat FRD.

The present study concluded that the timing of fungicide application is important in managing FRD incidence. The efficacy of these fungicides was higher when applied before the onset of rains in the last week of May, where 75%–87% FRD severity, 65%–72% FRD incidence, and 85%–92% CFNR were recorded (Mueller et al., 2017). However, the efficacy was relatively lesser when applied at the mid-monsoon and fag-end of the rainy season compared with that before the onset of the rains. The timing of fungicide application is a crucial factor in managing plant diseases for several reasons. Firstly, the effectiveness of a fungicide depends on its ability to reach the pathogen and stop its growth. The timing of application can impact this ability as the disease progresses and the pathogen spreads. For example, applying a fungicide at an early stage of the disease when the pathogen has not yet colonized the plant tissue can result in better control of the disease compared with applying it at a later stage when the pathogen has already established itself. Accordingly, our study showed that if the recommended fungicides are applied at an early stage of FRD colonization, the future buildup of disease inoculum can be greatly reduced.

Secondly, the timing of application also affects the cost-effectiveness of the fungicide. Applying a fungicide too early or too late can result in a lower efficacy and may require additional applications to achieve the same level of control. Therefore, choosing the right timing for fungicide applications can help to reduce the overall disease management cost. Lastly, the timing of application can also impact the safety and environmental impact of the fungicide. Some fungicides are more lethal at certain stages of the plant’s growth, and applying them at these stages can result in harmful effects on the environment and non-target organisms. In India, arecanut is largely grown in the coastal region where fishing is a major industry. This region also experiences heavy rainfall during the monsoon season. Therefore, the indiscriminate use of fungicides during heavy rainfall may lead to contamination of the environment, including fish ponds. Therefore, choosing the right timing for application can help to minimize the environmental impact of the fungicide.

The results from experiment 2 (EXPT2) showed that copper-based fungicides can reduce FRD severity by 10%–23.75% and FRD incidence by 25%–33%, which is consistent with the findings of Narayanaswamy et al. (2017). However, the efficacy of Bordeaux mixture was found to be lower (15%–30% reduction in FRD incidence) compared with the results reported by Gangadhara Naik et al. (2019), who observed a 35%–42% reduction in FRD incidence. When fungicides (copper-based, phenyl amid, and newer oomycete-specific) were applied prior to the onset of the monsoon season (in EXPT1), their efficacy was significantly higher.

In conclusion, our study confirmed that the timing of fungicide applications to control FRD under field conditions should be based on the timing of monsoon onset rather than on fruit/nut stages or arecanut phenology. The growers and researchers should therefore adopt a calendar-based approach (related to environmental conditions and therefore to the moment of infection of the pathogen) for fungicides instead of a phenology-based approach. Our results highlighted the understanding of FRD occurrence over the application timing of fungicides. Currently, most predictive models have been developed for controlling diseases on other hosts (Musa et al., 2007; Gourdain et al., 2011; Bondalapati et al., 2012), but there should be a focus on predicting FRD under field conditions. These models characterize the impact of weather variables on FRD and are mostly regression-based models obtained from various field data. Our study has confirmed the efficacy of oomycete-specific fungicides on FRD disease levels and CFNR by using GLLMM and integrating the effects of weather variables on FRD infection. The information generated from this study will be helpful for growers, stakeholders, policymakers, and the scientific community to select and apply fungicides in a timely manner to control FRD.

## Data availability statement

The datasets presented in this study can be found in online repositories. The names of the repository/repositories and accession number(s) can be found below: GenBank, ON999172.1.

## Author contributions

PB: conceptualization, design, investigation, data analysis, manuscript preparation. VH, HN, and SSr: conceptualization, methodology development, investigation, provision of the resources. SSh, SHT, and RTPP methodology, formal data analysis, critical revision of the manuscript. SRMS and RC: statistical analysis, interpretation of the data, manuscript—review, writing, and editing. All authors contributed to the article and approved the submitted version.
